# Association of SNPs of CD40 Gene with Multiple Sclerosis in Russians

**DOI:** 10.1371/journal.pone.0061032

**Published:** 2013-04-22

**Authors:** Ekaterina Alekseevna Sokolova, Nadezhda Alekseevna Malkova, Denis Sergeevich Korobko, Aleksey Sergeevich Rozhdestvenskii, Anastasia Vladimirovna Kakulya, Elena Vladimirovna Khanokh, Roman Andreevich Delov, Fedor Alekseevich Platonov, Tatyana Yegorovna Popova, Elena Gennadievna Aref′eva, Natalia Nikolaevna Zagorskaya, Valentina Mikhailovna Alifirova, Marina Andreevna Titova, Inna Vadimovna Smagina, Svetlana Alksandrovna El′chaninova, Anna Valentinovna Popovtseva, Valery Pavlovich Puzyrev, Olga Georgievna Kulakova, Ekaterina Yur'evna Tsareva, Olga Olegovna Favorova, Sergei Gennadievich Shchur, Natalia Yurievna Lashch, Natalia Fyodorovna Popova, Ekaterina Valerievna Popova, Evgenii Ivanovich Gusev, Aleksey Nikolaevich Boyko, Yurii Sergeevich Aulchenko, Maxim Leonidovich Filipenko

**Affiliations:** 1 Pharmacogenomics Group, Institute of Chemical Biology and Fundamental Medicine, Siberian Division, Russian Academy of Sciences, Novosibirsk, Russia; 2 Multiple Sclerosis Center, Novosibirsk Regional State Clinical Hospital, Novosibirsk, Russia; 3 Department of Neurology of Postgraduate Education, Omsk State Medical Academy, Omsk, Russia; 4 Omsk Regional Clinical Hospital, Omsk, Russia; 5 Budgetary Healthcare Facility of the Omsk Region, Clinical Diagnostic Center, Omsk, Russia; 6 Multiple Sclerosis Center, Republican Hospital No. 2 of Ministry of Health of the Republic of Sakha, Yakutsk, Russia; 7 Department of Neurology of Medical University, North-Eastern Federal University named after M.K. Ammosov, Yakutsk, Russia; 8 State Budget Institution of Healthcare “Kemerovo Regional Clinical Hospital”, Kemerovo, Russia; 9 Department of Neurology and Neurosurgery, Siberian State Medical University, Federal Agency for Health and Social Development, Tomsk, Russia; 10 Territorial Clinical Hospital, Barnaul, Russia; 11 Department of Nervous Diseases, Altai State Medical University, Barnaul, Russia; 12 Russian Academy of Medical Sciences Facility, Research Institute of Medical Genetics, Siberian Division, Russian Academy of Medical Sciences, Tomsk, Russia; 13 Department of Neurology and Neurosurgery, The Russian National Research Medical University named after N.I. Pirogov (RNRMU), Moscow, Russia; 14 Moscow Multiple Sclerosis Center (MMSC) at the 11th City Hospital of Moscow, Moscow, Russia; 15 Laboratory of Genetic Recombination and Segregation, Institute of Cytology and Genetics, Siberian Branch of the Russian Academy of Sciences, Novosibirsk, Russia; Kunming Institute of Zoology, Chinese Academy of Sciences, China

## Abstract

Multiple sclerosis (MS) is a serious, incurable neurological disease. In 2009, the ANZgene studies detected the suggestive association of located upstream of CD40 gene in chromosome 20q13 (p = 1.3×10^−7^). Identification of the causal variant(s) in the *CD40* locus leads to a better understanding of the mechanism underlying the development of autoimmune pathologies. We determined the genotypes of rs6074022, rs1883832, rs1535045, and rs11086996 in patients with MS (n = 1684) and in the control group (n = 879). Two SNPs were significantly associated with MS: rs6074022 (additive model C allele OR = 1.27, 95% CI = [1.12–1.45], *p* = 3×10^−4^) and rs1883832 (additive model T allele OR = 1.20, 95% CI = [1.05–1.38], *p* = 7×10^−3^). In the meta-analysis of our results and the results of four previous studies, we obtain the association *p*-value of 2.34×10^−12^, which confirmed the association between MS and rs6074022 at a genome-wide significant level. Next, we demonstrated that the model including rs6074022 only sufficiently described the association. From our analysis, we can speculate that the association between rs1883832 and MS was induced by LD, whereas rs6074022 was a marker in stronger LD with the functional variant or was the functional variant itself. Our results indicated that the functional variants were located in the upstream region of the gene *CD40* and were in higher LD with rs6074022 than LD with rs1883832.

## Introduction

Multiple sclerosis (MS) is a serious, incurable neurological disease. The etiology of MS is linked to various genetic and environmental factors. Fifteen genome-wide association studies (GWASs) on MS have been conducted so far, and more than 50 loci have been identified [1]–[15]. The Australia and New Zealand Multiple Sclerosis Genetics Consortium (ANZgene) conducted a GWAS in 2009 and confirmed a number of previously reported associations. These associations include *HLA-DR15* (*p* = 7.0×10^−184^), *CD58* (*p* = 9.6×10^−8^), *EVI5-RPL5* (*p* = 2.5×10^−6^), *IL2RA* (*p* = 7.4×10^−6^), *CLEC16A* (*p* = 1.1×10^−4^), *IL7R* (*p* = 1.3×10^−3^), and *TYK2* (*p* = 3.5×10^−3^). The ANZgene study also detected the suggestive association of two single nucleotide polymorphisms (SNPs) located upstream of *CD40* gene in chromosome 20q13 (rs6074022, *p* = 1.3×10^−7^; rs1569723, *p* = 2.9×10^−7^) [7]. An association between rs6074022 and MS was also identified in a GWAS based on a cohort from the United Kingdom [15]. However, in the meta-analysis reported in 2011, the association between SNP rs6074022 and MS did not reach the GWAS significance level (*p* = 4.91×10^−6^) [15].

Association with the *CD40* gene region has been implicated in a number of human autoimmune diseases such as systemic lupus erythematosus (SLE) [16], rheumatoid arthritis (RA) [17], Crohn's disease [18], and Grave's disease [19]. The participation of CD40 in autoimmune processes is clearly demonstrated in experimental animal models. A non-obese diabetic (NOD) mouse line is one of the most studied models of autoimmune diabetes. The disruption of CD40-CD40L interactions by antagonistic antibodies to CD40L prevents the development of diabetes in NOD mice, which confirms the critical function of the CD40-CD40L complex in the development of this disease [20].

In the mouse line K/BxN, RA spontaneously develops with many characteristics similar to the clinical course of RA in humans. CD40 knockout (K/BxN-CD40-/-) mice do not develop RA [21]. If a CIA mouse model of RA is treated with antagonistic antibodies such as CD40L mAb before the onset of collagen-induced arthritis, the mouse do not develop RA [22] or the disease decreases in severity [23]. However, if the therapy is started after the development of arthritis, no improvement occurs [25], [26].

CD40 contributes to the susceptibility to human autoimmune diseases, in which the B and T cell pathways play key roles. The role of CD40-CD40L interactions is identified in the development of type-1 diabetes. The CD40 signaling pathway induces the production of proinflammatory cytokines in the islet cells of primates and humans [24]. Increased expression of CD40L^+^ cells has been observed in the brains of patients with MS [25].

Also, the expression of CD40 in keratinocytes and endothelial cells in psoriatic plaques, as well as the increased expression of CD40L in the peripheral blood T cells [26], have been shown in patients with psoriatic arthritis. An elevated level of circulating CD40L has been found in patients with RA, SLE, and Sorgena syndrome during exacerbation [27]. In fact, the interaction between CD40 and CD40L triggers the immune response.

The potential association of the *CD40* region with MS is in accordance with the theory that MS has an autoimmune origin. Data from animal models also suggest similarities among the molecular mechanisms underlying the development of various immune disorders. Identification of the causal variant(s) in the *CD40* locus will lead to a better understanding of the mechanism underlying the development of autoimmune pathologies. This study aimed to replicate a previously reported association of rs6074022 with MS in a Russian population and to study the association of MS with some other SNPs of the *CD40* region.

## Materials and Methods

### Patients

Among a group of Russian-ethnicity patients with MS, 1684 people (1124 women and 560 men; mean age±SD = 36.7±11.2 years) were included based on the McDonald criteria for MS [28]. A total of 1176 patients had relapsing remitting MS (RRMS), 73 had primary progressive MS (PPMS), 403 had secondary-progressive MS (SPMS), and 32 patients had clinically isolated syndrome (CIS). The following centers were involved in the recruitment of patients: Moscow Multiple Sclerosis Center at the City Hospital No. 11, *n* = 508; Omsk Regional Clinical Hospital, *n* = 305; State Budget Institution of Healthcare “Kemerovo Regional Clinical Hospital”, *n* = 224; Department of Neurology and Neurosurgery, Siberian State Medical University, Federal Agency for Health and Social Development (Tomsk), *n* = 152; Territorial Clinical Hospital (Barnaul), *n* = 146; Novosibirsk Regional State Clinical Hospital, *n* = 248; and Republican Hospital No. 2 of the Ministry of Health of the Republic of Sakha, *n* = 101.

The control group included individuals (*n* = 879) without inflammatory diseases of the central nervous system living in Novosibirsk (*n* = 567), Barnaul (*n* = 118), Moscow (*n* = 112), Yakutsk (*n* = 60), and Omsk (*n* = 22). The group consisted of 346 men and 533 women (mean age±SD = 33.0±12.0 years).

This study was approved by the ethics committees of all participating centers. All participants signed a written informed consent.

### Selection of SNPs

**Figure 1:**
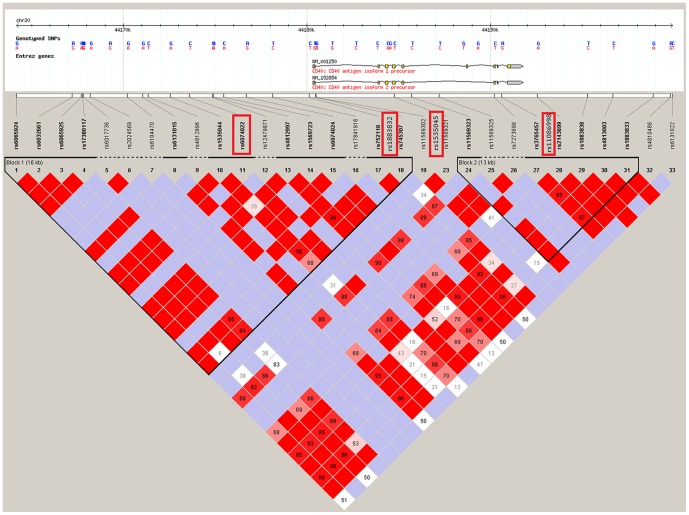
LD between the SNPs *CD40* gene. According to the result of the HapMap Project (v.3). union panel for Utah residents with Northern and Western European (CEU) and Toscans in Italy (TSI). SNPs from our study are shown in red frame.

The *CD40* region has two blocks of linkage disequilibrium (LD) according to the result of the *HapMap 3* panel for Utah residents of Northern and Western European descent and for Toscans in Italy (Fig. 1). In our study, we selected the following four SNPs: rs6074022 and rs1883832 from the first block of LD, rs11086998 from the second block, as well as rs1535045 from the space between the two blocks.

The two SNPs previously associated with MS lie in the first block of LD. SNP rs6074022 T->C was associated with MS at a genome-wide suggestive level (*p* = 1.3×10^−7^) [7]. SNP rs1883832 C->T is located in the −1 position relative to the start of the transcription of *CD40* gene. In previous candidate gene studies, the association of the minor allele T of SNP rs1883832 with a number of diseases has been examined. Such diseases include lymphoma [29], non-Hodgkins lymphoma [30], osteoporosis [31], multiple sclerosis [18], Crohn's disease [18], sporadic breast cancer [32], Behcet's syndrome [33], and rheumatoid arthritis [34].

In the second block, we selected SNP rs11086998 based on its potential function. The presence of minor G allele in rs11086998 leads to a proline-to-alanine substitution (P227A) in the intracellular region of CD40. The P227A site is 3 amino acid residues proximal to the TRAF6 binding site, and leds to increased TNF-α and IL-6 production in murine cell lines [35]. Notably, the rs11086998[G] allele has rather different frequencies in different ethnic groups, from 29% in Mexicans and South Americans to <2% in others. SNP rs1535045 was chosen to tag the gap between the two blocks of LD.

### Genotyping

DNA was extracted from venous blood using standard procedures, including selection and lysis of blood cells, the hydrolysis of proteins with proteinase K, DNA purification by extraction with phenol-chloroform, and the DNA precipitated with ethanol. Genotyping of SNPs rs6074022, rs1883832, rs1535045 and rs11086996 was performed by TaqMan real-time PCR (ICBFM SB RAS, Novosibirsk, Russia).

### Statistical data analysis

Tests for the Hardy-Weinberg equilibrium were performed using the DeFinetti program available from the website of the Institute of Human Genetics (Munich, Germany; http://ihg2.helmholtz-muenchen.de/cgi-bin/hw/hwa1.pl). Associations of genotype with the disease was studied using logistic regression analysis, as implemented in “glm” function of the R package for statistical analysis (www.r-project.org). The likelihood ratio test (LRT) was used to test the statistical hypotheses and the Akaike Information Criterion (AIC) was used to decide on “best model” describing the association of MS with SNPs. Meta-analysis and Q-test were carried out using the ‘rmeta' package for R (http://cran.r-project.org/web/packages/rmeta/rmeta.pdf). Haplotype analysis was carried out using the ‘haplo.stats' package for R (http://cran.r-project.org/web/packages/haplo.stats/haplo.stats.pdf). Results were considered statistically significant for all statistical calculations if *P*<0.05.

## Results

We determined the genotypes of rs6074022, rs1883832, rs1535045, and rs11086996 in patients with MS and in the control group (Table 1). The call rate was≥99.9% for all SNPs. Five patients were excluded from further analysis because of missing data in at least one locus. Genotypic distribution did not significantly deviate from the Hardy–Weinberg equilibrium expectations for all four studied SNPs in the MS and control groups. The Q-test for heterogeneity of the minor allele frequency between sub-samples from different cities did not show significant differences (Table S1).

**Table 1:** Logistic regression association results for SNPs of *CD40* gene with the development of MS.

SNP		Number of		risk allele	RAF	genotypic model: OR [95% CI], p-value, AIC	additive model: OR [95% CI], p-value, AIC	dominant model: OR [95% CI], p-value, AIC	recessive model: OR [95% CI], p-value, AIC
		Controls	Cases						
rs6074022	T/T	517	865	C	0.24	Reference	***1.27 [1.12***–***1.45] p = 0.0003***	*1.34 [1.14*–*1.58] p = 0.0004*	*1.39 [1.01*–*1.93] p = 0.04*
	T/C	307	671			*1.31 [1.10*–*1.55] p = 0.003*			
	C/C	55	143			*1.55 [1.12*–*2.16] p = 0.009*			
	HWE	0.30	0.43			AIC = 3284.3	***AIC = 3282.4***	*AIC = 3283.3*	*AIC = 3291.4*
rs1883832	C/C	532	927	T	0.22	Reference	***1.20 [1.05***–***1.38] p = 0.007***	*1.24 [1.05*–*1.47] p = 0.01*	1.31 [0.93–1.85] p = 0.12
	C/T	299	634			*1.22 [1.02*–*1.45] p = 0.03*			
	T/T	48	118			1.41 [0.99–2.01] p = 0.6			
	HWE	0.48	0.50			AIC = 3290.4	***AIC = 3288.4***	*AIC = 3289*	AIC = 3292.3
rs1535045	C/C	481	957	T	0.26	Reference	0.96 [0.84–1.09] p = 0.52	0.91 [0.77–1.07] p = 0.27	1.10 [0.79–1.53] p = 0.57
	C/T	342	605			0.89 [0.78–1.06] p = 0.18			
	T/T	56	117			1.05 [0.75–1.47] p = 0.78			
	HWE	0.64	0.11			AIC = 3295.6	AIC = 3295.3	AIC = 3294.5	AIC = 3295.4
rs11086996	C/C	852	1640	G	0.02	Reference	0.75 [0.46–1.23] p = 0.26	0.91 [0.77–1.07] p = 0.26	not applicable
	C/G	27	39			0.75 [0.46–1.23] p = 0.26			
	G/G	0	0			not applicable			
	HWE	0.64	0.63			AIC = 3294.4	AIC = 3294.4	AIC = 3294.4	not applicable

Abbreviations: 95% CI, 95% confidence interval; OR, odds ratio; NA, not applicable; RAF, risk allele frequency in control group; SNP, single-nucleotide polymorphism; HWE-p-value of exact test for deviation from Hardy-Weinberg equilibrium in groups; AIC–Akaike Information Criterion. Analysis was performed for four models: co-dominant, dominant, additive and recessive. Significant associations are shown in italic. The best model for each of significant associated SNPs is shown in bold.

Analysis of the associations of MS was performed using logistic regression under the additive, dominant, recessive, and genotypic (two degrees of freedom, 2df) models (Table 1). Two SNPs were significantly associated with MS: rs6074022 (additive model C allele OR = 1.27, 95% CI =  [1.12–1.45], *p* = 3×10^−4^) and rs1883832 (additive model T allele OR = 1.20, 95% CI = [1.05–1.38], *p* = 7×10^−3^). According to the Akaike information criterion (AIC) for each of these SNPs, the additive model was the best. Neither rs1535045 nor rs11086998 was associated with MS.

Additionally, we performed stratified analysis to exclude a potential confounding by genetic substructure in our study. Sub-samples from each city were assigned a stratum in the stratified analysis (Figure S1). The summary OR, its 95% confidence interval, significant level, and *p*-level of heterogeneity are shown in Table S2 for each SNP. The results of stratified analysis were in accordance with the association analysis in the entire group. Despite the smaller sample size (the Kemerovo and Tomsk groups were excluded in the meta-analysis because they contained only cases), we observed in the joint analysis that rs6074022 (*p* = 0.02) and rs1883832 (*p* = 4×10^−3^) were associated with MS. The risk alleles were the same as those found in the association analysis in the entire group.

We also analyzed the association of clinical sub-phenotypes of MS (RRMS, PPMS, SPMS, and CIS) with all SNPs (Table S3). After correction for multiple testing RRMS–the most common sub-type–was associated with rs6074022 (OR = 1.26, *p* = 0.0008) and with rs1883832 (OR = 1.24, *p* = 0.003).

We performed a haplotype analysis for rs6074022 and rs1883832. The haplotype frequency, OR, its 95% confidence interval, and significant level are shown in Table 2. Two haplotypes were associated with MS: rs6074022[C]-rs1883832[C] (OR = 2.38, 95% CI = 1.76–3.22, empirical *p* = 1.8×10^−8^) and rs6074022[T]-rs1883832[T] (OR = 2.68, 95% CI = 1.79–4.02, empirical *p* = 1.7×10^−6^).

**Table 2:** Analysis of association between MS and haplotypes at SNPs rs6074022 and rs1883832.

Rs6074022	Rs1883832	Frequency in Cases	Frequency in Control	Sample frequency	OR	95% C.I.	Empirical p-value
T	C	0.672391	0.746405	0.698116	reference	–	–
C*	T*	0.216465	0.208293	0.213949	1.19	1.03–1.38	0.02
T	T*	0.042618	0.016394	0.033314	2.68	1.79–4.02	1.7×10^−6^
C*	C	0.068526	0.028908	0.054620	2.38	1.76–3.22	1.8×10^−8^

Abbreviations: 95% CI, 95% confidence interval; OR, odds ratio; Sample frequency–haplotype frequency in MS and control groups together; empirical p-value–p-value of association haplotype with MS;

* -marked the risk allele from association analysis for each SNPs alone. Analysis was performed using logistic regression.

We estimated the LD between the studied SNPs of *CD40* gene (Figure S2). Relatively high values of *D*′ were detected in all SNP pairs. However, the *r* ^2^ between SNPs was weak at <0.1, except for rs6074022-rs1883832 (*r* ^2^ = 0.59).

We used the LRT to compare three models of association of rs6074022 and rs1883832 with MS: the general model (where we estimated the effects of genotypes of both SNPs rs1883832 and rs6074022) and two nested (effects estimated for only one SNP). According to the LRT, the nested model including rs6074022 only did not significantly differ from the more general model, including both SNPs rs6074022 and rs1883832 (*p* = 0.99), whereas the nested model including rs1883832 only was significantly worse than the general model (*p* = 0.01). The model including rs6074022 only was also the best according to the AIC (AIC = 3284.4, 3282.4, and 3288.4 for the general, rs6074022, and rs1883832 models, respectively). We conclude that the association of MS with the *CD40* locus can be described in terms of the involvement of rs6074022 only.

**Figure 2:**
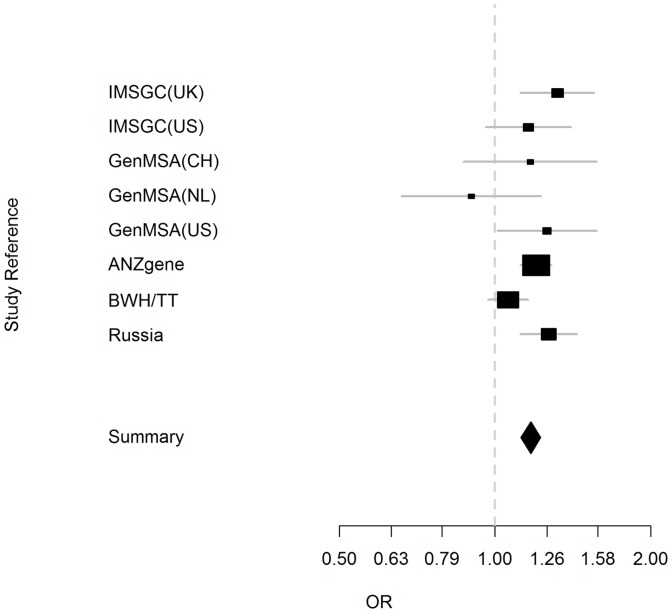
Meta-analysis of our results with previously published data on the association between rs6074022 and MS. Abbreviation: GenMSA (NL), GenMSA (US), GenMSA (CH), IMSGC (UK), IMSGC (US), BWH/TT, and ANZgene. In the meta-analysis the total OR for all studies was 1.17 (95% CI = 1.10–1.23) with a statistical significance of *p* = 2.24×10^−12^. The heterogeneity test (*Q*-test) did not find significant differences between the studies (*χ* ^2^ (7) = 12.16, *p* = 0.10).

We also performed a meta-analysis of our results with previously published data on the association between rs6074022 and MS: GenMSA (NL), GenMSA (US), GenMSA (CH) [1], IMSGC (UK), IMSGC (US) [3], BWH/TT [2], and ANZgene [7]. Table 3 summarizes the results of previous studies used for this meta-analysis. In the meta-analysis (Figure 2), the total OR for all studies was 1.17 (95% CI = 1.10–1.23) with a statistical significance of *p* = 2.24×10^−12^. The heterogeneity test (*Q*-test) did not find significant differences between the studies (*χ* ^2^ (7) = 12.16, *p* = 0.10). These data confirmed the association of marker locus rs6074022 and MS at a level of significance accepted for GWASs.

**Table 3:** Meta-analysis of association between rs6074022 C allele and MS.

Study ID	OR	95% C.I.	p-value	Case*	Control*	F:M ratio	Reference
IMSGC (UK)	1.32	[1.12–1.55]	1×10^−3^	449	2928	3.0∶1.0	[3]
IMSGC (US)	1.16	[0.96–1.42]	0.12	341	1679	3.2∶1.0	[3]
GenMSA (CH)	1.17	[0.87–1.58]	0.30	251	208	2.8∶1.0	[1]
GenMSA (NL)	0.90	[0.66–1.22]	0.51	225	228	2.9∶1.0	[1]
GenMSA (US)	1.26	[1.01–1.56]	0.04	477	425	3.1∶1.0	[1]
ANZgene	1.20	[1.12–1.28]	1.3×10^−7^	1616	1987	2.6∶1.0	[7]
BWH/TT	1.06	[0.97–1.17]	0.20	2186	4689	2.6∶1.0	[2]
Russia	1.27	[1.12–1.45]	0.0003	1679	879	2.0∶1.5	our study
**Summary**	**1.16**	**[1.12**–**1.23]**	2.2×10^−12^	**7224**	**13023**		

Study ID: IMSGC (UK)–International Multiple Sclerosis Genetics Consortium United Kingdom; IMSGC (US)–International Multiple Sclerosis Genetics Consortium United States; GenMSA (CH)–Switzerland, Basel; GenMSA (NL)–The Netherlands, Amsterdam; GenMSA (US)–United States, San Francisco; ANZgene–Australia and New Zealand Multiple Sclerosis Genetics Consortium; BWH/TT–Brigham & Woman's Hospital, Therapeutic Trials. F–female, M–male.

* -Analyzed cases and controls per data set after quality control.

Results of our meta-analysis are shown in bold.

## Discussion

CD40-CD40L is reportedly a common link in the pathogenesis of autoimmune diseases. This hypothesis is supported by the established role of the CD40–CD40L interaction in the development of several autoimmune conditions in animal models [20]–[23] and by the association of CD40 SNPs with the risk for a number of autoimmune diseases [16]–[19]. Moreover, the successfully completed Phase 1 of the clinical trial of SLE treatment by CD40L (http://www.biogenidec.com/research_product_pipeline.aspx?ID=5778) provides evidence of the important role of the CD40–CD40L interaction in the pathogenesis of SLE. The CD40–CD40L interaction is known to result in the switch to antigen-specific Th2 type response [36]. Therefore, the CD40–CD40L complex is an extremely attractive and promising target for the development of drugs for suppressing autoimmune attack. However, for the successful creation of drugs, the molecular mechanism underlying the initiation of autoimmune inflammation through CD40–CD40L in humans must be studied.

In this work, we aimed to replicate in the Russian population the previously reported association of rs6074022[C] with MS [7]. We observed a statistically significant association of the allele rs6074022[C] with the development of MS (per C allele OR = 1.27, CI = 1.12–1.45, *p* = 3×10^−4^). Our results were in accordance with those of ANZgene (Table S4). The minor allele C of rs6074022 had a similar frequency both in the case and control groups. Also, the minor allele was the risk-associated allele in both studies. SNP rs1883832 was less significantly associated with MS than rs6074022 in both studies.

In a previous meta-analysis of GWASs, rs6074022 has been implicated at a suggestive level at most [37]. In the meta-analysis of our results and the results of four previous studies, we obtain the association *p*-value of 2.24×10^−12^, which confirmed the association between MS and rs6074022 at a genome-wide significant level.

Our findings of the association between MS and *CD40* gene are in accordance with the hypothesis of the autoimmune nature of MS. However, the molecular mechanism underlying the involvement of SNPs of *CD40* gene in the autoimmune processes was unclear for MS and other autoimmune diseases. The first step in solving this problem is to determine functional SNPs in the gene.

Haplotype analysis revealed two haplotypes associated with MS: rs6074022[C]-rs1883832[C] (OR = 2.38, 95% CI = 1.76–3.22, empirical *p* = 1.8×10^−8^) and rs6074022[T]-rs1883832[T] (OR = 2.68, 95% CI = 1.79–4.02, empirical *p* = 1.7×10^−6^). Each haplotype contains one of the “risk” alleles as identified in single-SNP analyses. Interestingly, the haplotype containing both risk alleles was not associated with MS in our analysis. We can speculate that this finding is consistent with the hypothesis of two functional polymorphisms located close to the marker SNPs, one increasing the risk of developing MS whereas the second being protective.

In our study, both SNPs rs6074022 and rs1883832 were significantly associated with MS (per C allele OR = 1.27, CI = 1.12–1.45, *p* = 3×10^−4^; per T allele OR = 1.20, CI = 1.05–1.38, *p* = 7×10^−3^). We also demonstrated that the model including rs6074022 only sufficiently described the association. A large proportion of polymorphisms located in the same block of LD with a causal variant are likely to show an association with a disease. However, the polymorphisms that are in greater LD with the functional variant should on average have a stronger association. From our analysis, we can speculate that the association between rs1883832 and MS is induced by LD, whereas rs6074022 is a marker in stronger LD with the functional variant or is the functional variant itself. We may speculate that the functional variant(s) is likely to be located in the upstream region of the gene *CD40* and is in higher LD with rs6074022 than with rs1883832.

## Summary

Our results confirmed the association of SNP rs6074022 with the risk of MS development. Our estimates suggeste that the functional variant(s) is located in the upstream region of the gene. Further empirical studies are required to find the functional variant.

## Supporting Information

Figure S1 Stratified analysis. Stratified analysis for rs6074022., B. Stratified analysis for rs1883832, C. Stratified analysis for rs1535045, D. Stratified analysis for rs1186998. Abbreviations: Nsk–Novosibirsk.
(TIF)

Figure S2 LD between the studied SNPs of CD40 gene.
(TIF)

Table S1 Minor allele frequency in control sub-groups from different cities.
(DOCX)

Table S2 Results of stratified analysis of association between MS and SNPs from CD40 gene. Significant associations are shown in italic and bold. Abbreviations: 95% CI, 95% confidence interval; OR, odds ratio; NA, not applicable; Heterogeneity p-value-p-value of test of heterogeneity (Q-test).
(DOCX)

Table S3 Analysis of association between the clinical sub-phenotypes of MS with SNPs. Abbreviations: OR, odds ratio, RRMS-relapsing remitting multiple sclerosis, PPMS-primary progressive multiple sclerosis, SPMS-secondary-progressive multiple sclerosis, CIS-clinically isolated syndrome. Significant association are shown in bold.
(DOCX)

Table S4 Compare the results of our study with one of ANZgene. RAF–risk allele frequency, OR–odds ratio, p-value–significant level, G* is correspond to C allele in our study.
(DOCX)
