# Supplementary figures and images for: Association of SNPs of CD40 Gene with Multiple Sclerosis in Russians
Source: PLoS One. 2013 Apr 22;8(4):e61032. doi: 10.1371/journal.pone.0061032 (PMC3632563; doi:10.1371/journal.pone.0061032)

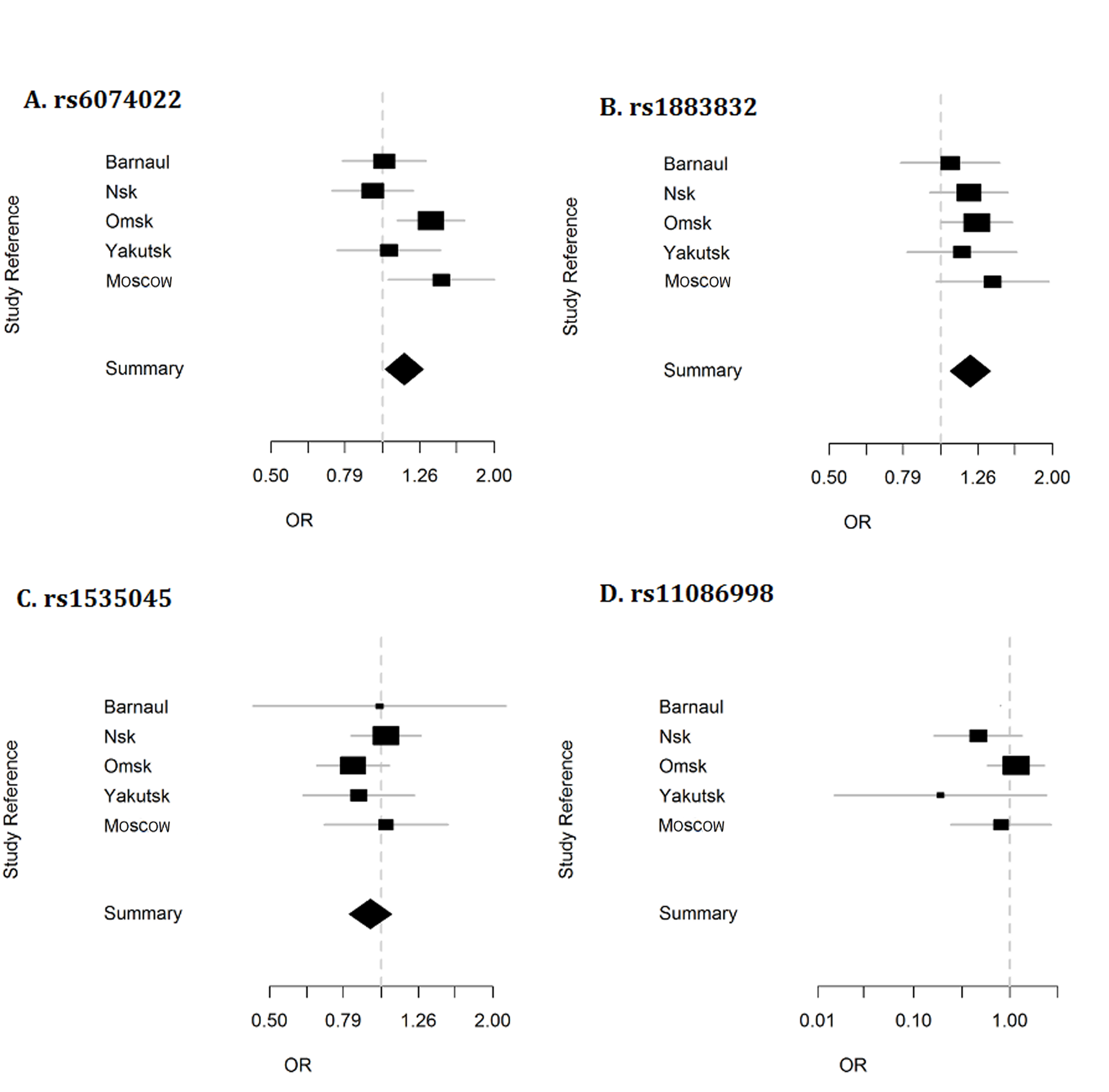

Supplement: Figure S1 — Stratified analysis. Stratified analysis for rs6074022., B. Stratified analysis for rs1883832, C. Stratified analysis for rs1535045, D. Stratified analysis for rs1186998. Abbreviations: Nsk–Novosibirsk. (TIF) [file pone.0061032.s001.tif]

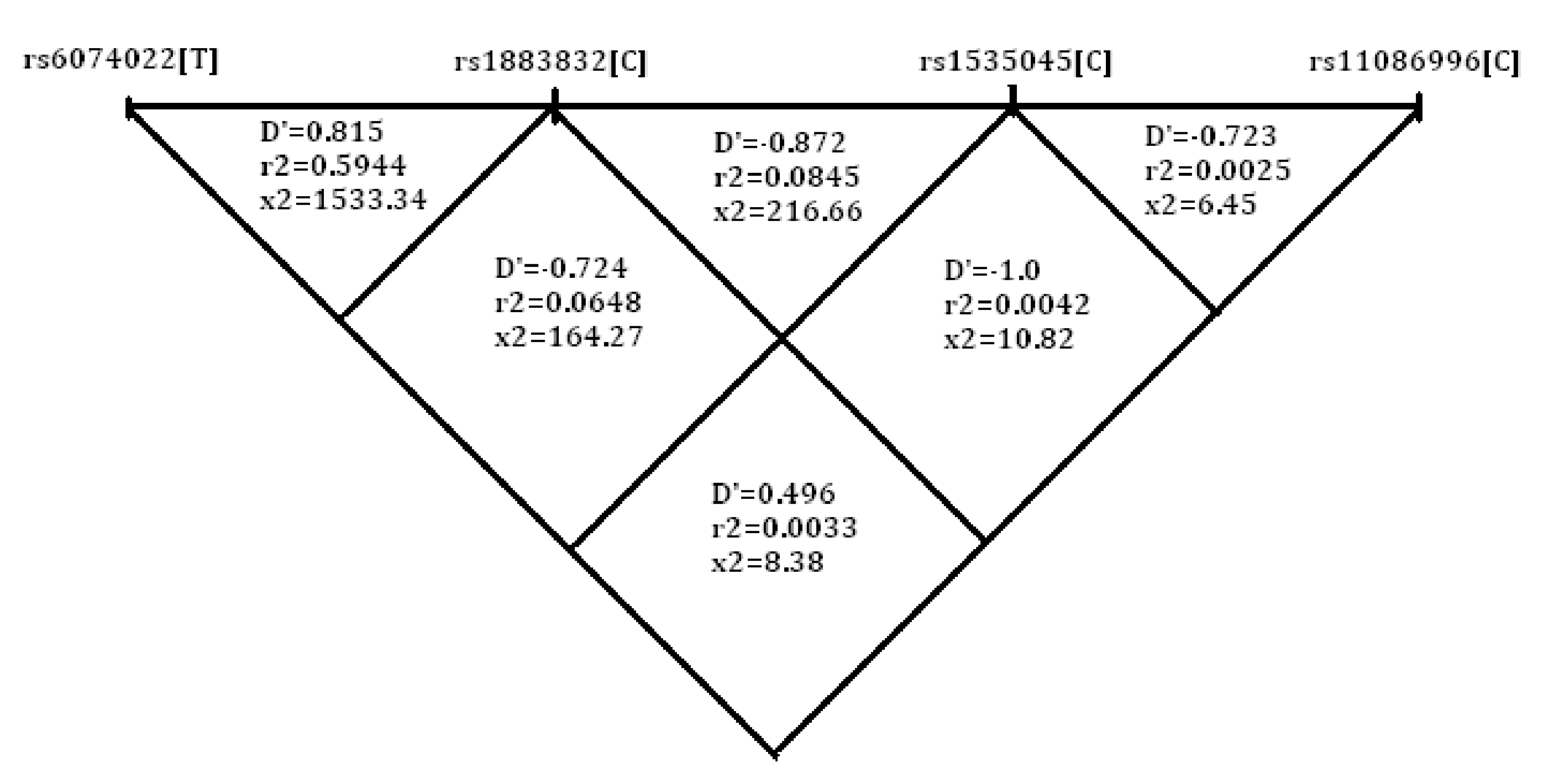

Supplement: Figure S2 — LD between the studied SNPs of CD40 gene. (TIF) [file pone.0061032.s002.tif]
