# Supplementary material for: Association of SNPs of CD40 Gene with Multiple Sclerosis in Russians
Source: PLoS One. 2013 Apr 22;8(4):e61032. doi: 10.1371/journal.pone.0061032 (PMC3632563; doi:10.1371/journal.pone.0061032)
Supplement: Table S1 — Minor allele frequency in control sub-groups from different cities. (DOCX) [file pone.0061032.s003.docx]

**Table S1. Minor allele frequency in control sub-groups from different cities**

| Control groups of cities’ cohorts | Minor allele frequency | | | |
| --- | --- | --- | --- | --- |
|  | rs6074022 | rs1883832 | rs1535045 | rs11086998 |
| Novosibirsk (n=576) | 0.24 | 0.23 | 0.26 | 0.02 |
| Barnaul (n=118) | 0.28 | 0.25 | 0.26 | 0.02 |
| Moscow (n=112) | 0.22 | 0.20 | 0.25 | 0.01 |
| Yakutsk (n=60) | 0.21 | 0.29 | 0.30 | 0.00 |
| Omsk (n=22) | 0.24 | 0.28 | 0.28 | 0.00 |
| Heterogenity (Q-test, p-value) | 0.55 | 0.36 | 0.89 | 0.42 |
