# Supplementary material for: Association of SNPs of CD40 Gene with Multiple Sclerosis in Russians
Source: PLoS One. 2013 Apr 22;8(4):e61032. doi: 10.1371/journal.pone.0061032 (PMC3632563; doi:10.1371/journal.pone.0061032)
Supplement: Table S2 — Results of stratified analysis of association between MS and SNPs from CD40 gene. Significant associations are shown in italic and bold. Abbreviations: 95% CI, 95% confidence interval; OR, odds ratio; NA, not applicable; Heterogeneity p-value-p-value of test of heterogeneity (Q-test). (DOCX) [file pone.0061032.s004.docx]

**Table S2. Results of stratified analysis of association between MS and SNPs from CD40 gene.**

Significant associations are shown in italic and bold. Abbreviations: 95% CI, 95% confidence interval; OR, odds ratio; NA, not applicable; Heterogeneity p-value - p-value of test of heterogeneity (Q-test).

| SNP | OR | 95% C.I. | p-value | Heterogeneity p-value |
| --- | --- | --- | --- | --- |
| rs6074022 | ***1.14*** | ***1.02-1.29*** | ***0.02*** | 0.09 |
| rs1883832 | ***1.20*** | ***1.06-1.36*** | ***0.004*** | 0.83 |
| rs1535045 | 0.94 | 0.82-1.09 | 0.33 | 0.73 |
| rs11086998 | 0.82 | NA | NA | 0.50 |
