# Supplementary material for: Association of SNPs of CD40 Gene with Multiple Sclerosis in Russians
Source: PLoS One. 2013 Apr 22;8(4):e61032. doi: 10.1371/journal.pone.0061032 (PMC3632563; doi:10.1371/journal.pone.0061032)
Supplement: Table S3 — Analysis of association between the clinical sub-phenotypes of MS with SNPs. Abbreviations: OR, odds ratio, RRMS-relapsing remitting multiple sclerosis, PPMS-primary progressive multiple sclerosis, SPMS-secondary-progressive multiple sclerosis, CIS-clinically isolated syndrome. Significant association are shown in bold. (DOCX) [file pone.0061032.s005.docx]

**Table S3. Analysis of association between the clinical sub-phenotypes of MS with SNPs.**

Abbreviations: OR, odds ratio, RRMS - relapsing remitting multiple sclerosis, PPMS - primary progressive multiple sclerosis, SPMS - secondary-progressive multiple sclerosis, CIS - clinically isolated syndrome. Significant association are shown in bold.

|  | **rs6074022** | | | | | |
| --- | --- | --- | --- | --- | --- | --- |
|  | TT | TC | CC | Risk allele | OR_c_ | p-value |
| RRMS | 605 | 466 | 100 | C | ***1.26*** | ***0.0008*** |
| PPMS | 36 | 32 | 5 |  | 1.24 | 0.18 |
| SPMS | 208 | 159 | 36 |  | ***1.28*** | ***0.009*** |
| CIS | 16 | 14 | 2 |  | 1.20 | 0.42 |
| **Total** | **865** | **671** | **143** |  | **1.26** | **0.0003** |
| **Control** | **517** | **307** | **55** |  |  |  |
|  | **Rs1883832** | | | | | |
|  | CC | CT | TT | Risk allele | OR_c_ 95%C.I. | p-value |
| RRMS | 637 | 445 | 89 | T | ***1.24*** | ***0.003*** |
| PPMS | 39 | 31 | 3 |  | 1.11 | 0.43 |
| SPMS | 237 | 142 | 24 |  | 1.06 | 0.54 |
| CIS | 14 | 16 | 2 |  | 1.48 | 0.10 |
| **Total** | **927** | **634** | **118** |  | **1.20** | **0.007** |
| **Control** | **532** | **299** | **48** |  |  |  |
|  | **Rs1535045** | | | | | |
|  | CC | CT | TT | Risk allele | OR_c_ 95%C.I. | p-value |
| RRMS | 656 | 434 | 81 | T | 0.99 | 0.79 |
| PPMS | 46 | 23 | 4 |  | 0.80 | 0.22 |
| SPMS | 234 | 139 | 30 |  | 0.98 | 0.54 |
| CIS | 21 | 9 | 2 |  | 0.79 | 0.32 |
| Total | 957 | 605 | 117 |  | 0.98 | 0.51 |
| Control | 481 | 342 | 56 |  |  |  |
|  | Rs11086998 | | | | | |
|  | CC | CG | GG | Risk allele | OR_c_ 95%C.I. | p-value |
| RRMS | 1151 | 20 | 0 | G | 0.55 | 0.04 |
| PPMS | 72 | 1 | 0 |  | 0.44 | 0.40 |
| SPMS | 385 | 18 | 0 |  | 1.48 | 0.21 |
| CIS | 32 | 0 | 0 |  | 2.10 | 0.31 |
| Total | 1640 | 39 | 0 |  | 0.75 | 0.26 |
| Control | 852 | 27 | 0 |  |  |  |
