# Supplementary material for: Association of SNPs of CD40 Gene with Multiple Sclerosis in Russians
Source: PLoS One. 2013 Apr 22;8(4):e61032. doi: 10.1371/journal.pone.0061032 (PMC3632563; doi:10.1371/journal.pone.0061032)
Supplement: Table S4 — Compare the results of our study with one of ANZgene. RAF–risk allele frequency, OR–odds ratio, p-value–significant level, G* is correspond to C allele in our study. (DOCX) [file pone.0061032.s006.docx]

**Table S4. Compare the results of our study with one of ANZgene.**

**RAF – risk allele frequency, OR – odds ratio, p-value – significant level, G* is correspond to C allele in our study**

|  | RAF cases | RAF controls | p-value | OR | Risk allele |
| --- | --- | --- | --- | --- | --- |
| rs6074022 (ANZgene) | 0.280 | 0.247 | 1.3 x 10^-7^ | 1.20 | G* |
| rs6074022 (our study) | 0.285 | 0.237 | 3 x 10^-4^ | 1.27 | C |
| rs1883832 (ANZgene) | ND | ND | 2.2 x 10^-4^ | ND | ND |
| rs1883832 (our study) | 0.224 | 0.259 | 7 x 10^-3^ | 1.20 | T |
